# Adjusting sowing date improves the photosynthetic capacity and grain yield by optimizing temperature condition around flowering of summer maize in the North China Plain

**DOI:** 10.3389/fpls.2022.934618

**Published:** 2022-08-08

**Authors:** Dong Guo, Chuanyong Chen, Xiangling Li, Rui Wang, Zaisong Ding, Wei Ma, Xinbing Wang, Congfeng Li, Ming Zhao, Ming Li, Baoyuan Zhou

**Affiliations:** ^1^Key Laboratory of Crop Physiology and Ecology, Ministry of Agriculture and Rural Affairs, Institute of Crop Science, Chinese Academy of Agricultural Sciences, Beijing, China; ^2^College of Agriculture, Northeast Agricultural University, Harbin, China; ^3^Maize Research Center, Beijing Academy of Agriculture and Forestry Sciences, Beijing, China; ^4^Hebei Key Laboratory of Crop Stress Biology, College of Agronomy and Biotechnology, Hebei Normal University of Science and Technology, Qinhuangdao, China

**Keywords:** summer maize, sowing date, temperature conditions, photosynthetic capacity, grain yield

## Abstract

Adjusting the sowing date to optimize temperature conditions is a helpful strategy for mitigating the adverse impact of high temperature on summer maize growth in the North China Plain (NCP). However, the physiological processes of variation in summer maize yield with sowing date-associated changes in temperature conditions around flowering remain to be poorly understood. In this study, field experiments with two maize varieties and three sowing dates (early sowing date, SD1, 21 May; conventional sowing date, SD2, 10 June; delay sowing date, SD3, 30 June) were conducted at Xinxiang of Henan Province in 2019 and 2020. Early sowing markedly decreased the daily mean temperature (*T*_mean_), maximum temperature (*T*_max_), and minimum temperature (*T*_min_) during pre-silking, while delay sowing markedly decreased those temperatures during post-silking. Under these temperature conditions, both varieties under SD1 at 12-leaf stage (V12) and silking stage (R1) while under SD3 at R1 and milking stage (R3) possessed significantly lower malondialdehyde (MDA) content in leaf due to higher activities of superoxide dismutase (SOD), peroxidase (POD), and catalase (CAT) compared to SD2. Therefore, SD1 at V12 and R1 stages and SD3 at R1 and R3 stages for both varieties showed significantly higher photosynthetic capacity, including higher SPAD, *F*_v_*/F*_m_, *P*_n_, *T*_r_, and *G*_s_, which promoted greater pre-silking dry matter (DM) accumulation for SD1 to increase the kernel number, and promoted greater post-silking DM accumulation for SD3 to increase the kernel weight, eventually increased the grain yield of SD1 and SD3 compared to SD2. Results of regression analysis demonstrated that *T*_mean_, *T*_max_, and *T*_min_ values from V12 to R1 stages lower than 26.6, 32.5, and 20.3°C are necessary for improving the kernel number, while *T*_mean_, *T*_max_, *T*_min_, and accumulated temperature (AT) values from R1 to R3 stages lower than 23.2, 28.9, 17.3, and 288.6°C are necessary for improving the kernel weight. Overall, optimal temperature conditions around flowering can be obtained by early (21 May) or delay (30 June) sowing to improve the kernel number or kernel weight due to improved photosynthetic capacity, eventually increasing the grain yield of summer maize in the NCP.

## Introduction

Maize (*Zea mays* L.) is one of the most important cereal crops in China and plays an important role in ensuring food security. The North China Plain (NCP) is one of the major maize production areas in China, accounting for 35% of the cultivated maize and 40% of the grain yield for maize production across the whole country (China Agricultural Yearbook, 2016). However, the climate has become warmer and warmer across the northern part of China in recent years (Tao et al., 2006; Liu et al., 2012), and the frequency of extreme high-temperature events has increased (Xiao et al., 2016), which could result in more than 15% decrease for maize yield or even no harvest (Chen et al., 2011; Liu et al., 2013).

In the NCP, summer maize is commonly planted immediately after winter wheat annually in the winter wheat-summer maize cropping system. From 2013 to 2018, the summer maize suffered from high-temperature stress yearly, with the duration extending from 10 to 30 days, and the temperature in most areas reached above 35°C, even reached over 40°C in some areas (Hua et al., 2020). Furthermore, the high-temperature stress commonly occurs in late July to early August, which is around the flowering stage of summer maize (Xu et al., 2021). Generally, the crop yield reduction promoted by the high-temperature stress around the flowering stage has been associated with a decrease in grain number and weight (Prasad et al., 2015). However, the damages to reproductive organs and losses of grain yield in maize vary with changing stages that high-temperature stress occurs (Lizaso et al., 2018). High-temperature stress (≥35°C) occurs during the maize flowering stage and results in a significant decrease of grain number by affecting pollen and silks activity, pollination, and fertilization, which is considered to be the main reason for maize yield reduction under high-temperature stress (Ehsan et al., 2015; Yan et al., 2018; Shao et al., 2021). Cicchino et al. (2010) and Rattalino et al. (2014) also found that the grain abortion caused by high-temperature stress (≥35°C) occurs after pollination and fertilization has a great effect on the grain number per ear of maize. The decrease of grains per ear could be over 88% under severe high-temperature stress (Suwa et al., 2010; Rattalino et al., 2014). Moreover, high-temperature stress during the grain filling stage could lower the grain filling rate and period, resulting in insufficient assimilate supply to grain, and then reduce maize grain weight and yield (Tao et al., 2016; Zhou et al., 2017). Therefore, for ensuring sustainable maize production in the NCP, it is essential to improve the understanding of summer maize grain yield responses to varying temperature conditions around flowering and explore suitable management practices to counteract the adverse effects of high-temperature stress on maize growth.

Adjusting the sowing date is an effective strategy to mitigate the adverse effects of climatic factors by optimizing the climate conditions during the crop growth period (Santos et al., 2017; Coelho et al., 2021; Deng et al., 2022; Li et al., 2022). Our previous study has demonstrated that sowing date-associated variation in temperature was the primary factor that influenced maize grain yield in the NCP (Zhou et al., 2016, 2017). However, under the variation of temperature conditions around flowering stage associated with sowing dates, the physiological determinants of summer maize yield changes and the quantitative relationship between maize yield traits and temperatures around the flowering stage remain to be poorly understood. Therefore, a 2-year field experiment with two maize varieties and three sowing dates was conducted in NCP. The purpose of this study was to (a) determine the effect of early and delay sowing date on the temperature conditions around the maize flowering stage; (b) evaluate the effects of temperature conditions around the flowering stage on the pre- or post-silking dry matter (DM) accumulation, photosynthetic parameters, anti−oxidative properties, grain yield, and yield components of maize; and (c) quantify the relationship between maize yield traits and temperatures around the flowering stage.

## Materials and methods

### Site description

Two field experiments were conducted in 2019 and 2020 at the Xinxiang Experimental Station of Chinese Academy of Agricultural Sciences (35°11inxiang Ex°48i08E), Xinxiang County, Henan Province, China. This area is a warm temperate continental monsoon climate, with annual average temperature of 14°, accumulated temperature (AT) above 10°C of 4,647°C, sunshine hours of 2,324 h, and precipitation of 573 mm. The average daily mean temperature and effective AT above 10°C during the maize growing season were 25 and 1,900°C, respectively. The International Soil Science Society (ISSS) Classification was used on the soil of this experimental field. In the 0–40 cm plow layer, the content of organic matter was 12.6 g kg^–1^, the content of available nitrogen was 62.2 mg kg^–1^, the content of available phosphorus was 16.7 mg kg^–1^, the content of available potassium was 109.8 mg kg^–1^, and the pH value was 8.1.

### Experimental design and management

A completely randomized block design with three replications was used in this experiment. The treatments were done on three sowing dates, namely, 21 May (early sowing), 10 June (conventional sowing), and 30 June (delay sowing), which were called SD1, SD2, and SD3, respectively. Maize hybrids ZD958 and XY335, which were widely planted in this area, were used in this study. The previous crop winter wheat was planted on 31 October and harvested on 20 May. After wheat harvest, maize was planted at a density of 60,000 plants ha^–1^, with 0.6 m row spacing. Each plot was 15 m long and 4.8 m wide and consisted of 8 rows. Experimental plots were irrigated prior to sowing seeds, and basal fertilizer was applied at the rates of 130 kg N ha^–1^, 120 kg P_2_O_5_ ha^–1^, and 90 kg K_2_O ha^–1^. Additional nitrogen fertilizer (120 kg N ha^–1^) was top-dressed at the beginning of the jointing stage. The amount of fertilizer applied was based on the existing levels of N, P, and K (determined from soil tests) to ensure that there was no nutrient deficiency. All the experimental plots were well managed, and no obvious water stress, diseases, or insect pests were found during the growing period.

### Weather data

The meteorological data [daily mean temperature (*T*_mean_), daily maximum temperature (*T*_max_), daily minimum temperature (*T*_min_), precipitation, and sunshine hours] during the experimental periods in 2019 and 2020 were provided by the Chinese Meteorological Administration (2020).

The effective AT was obtained by summing up the mean daily temperatures during the period in which the mean daily temperature was above a base temperature in each day (Yan et al., 2011).


(1) AT(Co)=Σ(T-meanT)0×growthduration

where *T*_0_ is the base temperature (10°C for maize).

The solar radiation was calculated using the equation (Gao et al., 2018):


(2) SolarradiationQ=Q(a+bS/S)00

where *Q* is the total solar radiation, *Q*_0_ is the astronomical radiation, *S* is the actual sunshine hours, *S*_0_ is the possible sunshine hours, *S*/*S*_0_ is the proportion of sunshine, and *a* and *b* are correction coefficients.

### Crop sampling and measurements

The date was recorded when more than 50% of the maize plants in each plot reached the following stages: the emergency stage (VE), 6-leaf stage (V6), 12-leaf stage (V12), silking stage (R1), milking stage (R3), and physiological maturity (R6).

Green leaf areas of the sampled plants were measured at V6, V12, R1, R3, and R6 stages. For each leaf, the length and maximum width were recorded, and the leaf area was computed based on the following formula (Tian et al., 2018):


(3) Expanded⁢leaf⁢area=leaf⁢length×maximum⁢width×0.75

(4) Rolled⁢leaf⁢area=leaf⁢length×maximum⁢width×0.5

(5) Leaf⁢area=Expanded⁢leaf⁢area+Rolled⁢leaf⁢area

Leaf area index (LAI) was calculated using the following formula:


LAI=leafarea(mplant2)-1×plantdensity(plantsha)-l

(6) /10,000(mha2)-h

Plant samples were collected to determine DM content at V6, V12, R1, R3, and R6 stages. Three adjacent plants in each row were sampled randomly from each plot. The sampled plants were dried at 105°C for 30 min and then at 70°C to maintain constant moisture content before being weighed.

The SPAD-502 chlorophyll analyzer was used to determine the SPAD value of 15 marked ear leaves for each plot at V12, R1, and R3 stages, with the determination method referring to the literature (Earl and Tollen, 1997).

The photosynthetic parameters, such as photosynthetic rate (*P*_n_), transpiration rate (*T*_r_), and stomatal conductance (*G*_s_), of 15 marked ear leaves for each plot were measured between 10:00 and 12:00 on sunny days at V12, R1, and R3 stages using a Li-6400 photosynthesis system (Li-Cor Inc., Lincoln, NB, United States). The photosynthetically active radiation, CO_2_ concentration in the leaf chamber, and leaf temperature were set at 1,800 μmol m^–2^ s^–1^, 400 μmol mol^–1^, and 30 ± 4°C, respectively, and the relative humidity was 50–60%.

Chlorophyll fluorescence parameters of the middle part of 15 ear leaves for each plot were determined with pocket PEA between 10:00 and 12:00 at V12, R1, and R3 stages on sunny days using a Li-6400 photosynthesis system. After a 20-min dark adaptation at the central region of a leaf using black leaf clips, the initial fluorescence (*F*_0_) and maximum fluorescence (*F*_m_) were determined. Maximal photochemical efficiency of PSII is given as follows:


(7) F/vF=m(F-mF)0/Fm

Five marked ear leaves for each plot were sampled at V12, R1, and R3 stages. The required enzyme solution for measuring the activity of superoxide dismutase (SOD), peroxidase (POD), and catalase (CAT) was extracted according to Bao et al. (2022). The SOD activity was recorded at 560 nm and defined as the amount of SOD required to produce a 50% inhibition of reduction of nitroblue tetrazolium (NBT); the POD activity was defined as the increase in absorbance of every 30 s at 470 nm; the CAT activity was assayed as a decrease in absorbance at 240 nm for 1 min, followed by the decomposition of hydrogen peroxide (H_2_O_2_). The content of malondialdehyde (MDA) was measured with thiobarbituric acid (TBA).

At harvest, 36 m^2^ of the crop area was harvested manually from the four center rows in each plot. The ears were counted at harvest from the four center rows in each plot to determine the number of ears per hectare. The 1,000-kernel weight was calculated as the average of three random samples of 500 kernels. The kernel number was recorded as the mean kernel number of 10 ears from each replication. Grain yield was calculated at 14% moisture content.

### Statistical analyses

Data preparation was performed using Microsoft Excel 2016. Grain yield, kernel number, kernel weight, photosynthetic parameters, MDA, SOD, POD, and CAT were subjected to a two-way ANOVA with sowing date and variety as fixed effects, using the general linear model of SPSS 20.0 (SPSS Institute, Inc.). DM was subjected to repeated measure analyses with sowing date and sampling stage (repeated measurement) as fixed effects. Residual normality was tested using quantile-quantile plots, while the variance homogeneity was obtained by Levene’s test. Means were compared using the least significant difference (LSD) test at a 5% level of probability. Nonlinear regression analysis was performed by using SPSS.

## Results

### Weather conditions and phenological stages

As shown in Table 1, the daily *T*_mean_, *T*_max_, *T*_min_, effective AT, and *R*_a_ during the maize growth period varied by sowing date. Early sowing (SD1) markedly decreased the values of *T*_mean_, *T*_max_, and *T*_min_ during pre-silking, while delay sowing (SD3) markedly decreased the values of *T*_mean_, *T*_max_, *T*_min_, and AT during post-silking for both varieties in 2 years. Compared to SD2, *T*_mean_, *T*_max_, *T*_min_, and AT averagely decreased by 11.8, 10.5, 12.4, and 16.3% from V12 to R1 stages under SD1, while those values averagely decreased by 13.6, 15.6, 25.7, and 24.6% from R1 to R3 stages under SD3, respectively. Different from temperature trends, early sowing increased the accumulated radiation during sowing to V12, R1 to R3, and R3 to R6 stages, whereas delay sowing decreased them. Compared to SD2, SD1 increased the accumulated radiation averagely by 12.2, 10.9, and 8.5%, while SD3 decreased the accumulated radiation averagely by 7.9, 3.4, and 2.7% during sowing to V12, R1 to R3, and R3 to R6 stages, respectively.

**TABLE 1:** Daily mean temperature (*T*_mean_), maximum temperature (*T*_max_), daily minimum temperature (*T*_min_), effective accumulated temperature (AT), and accumulated radiation (*R*_a_), during the maize growth stage in 2019 and 2020.

Sowing date	Growth stage	2019										2020									
			
		ZD 958					XY 335					ZD 958					XY 335				
					
		*T*_mean_ (^o^C)	*T*_max_ (^o^C)	*T*_min_ (^o^C)	AT (^o^C)	*R*_a_ (MJ m^–2^)	*T*_mean_ (^o^C)	*T*_max_ (^o^C)	*T*_min_ (^o^C)	AT (^o^C)	*R*_a_ (MJ m^–2^)	*T*_mean_ (^o^C)	*T*_max_ (^o^C)	*T*_min_ (^o^C)	AT (^o^C)	*R*_a_ (MJ m^–2^)	*T*_mean_ (^o^C)	*T*_max_ (^o^C)	*T*_min_ (^o^C)	AT (^o^C)	*R*_a_ (MJ m^–2^)
SD1	Sowing to V12	25.6	31.3	19.4	715.2	864.4	25.5	31.3	19.2	702.4	877.7	25.5	31	20.9	710.9	852.8	25.4	30.8	20.7	707.9	877.1
	V12 to R1	26.7	32.5	20.4	207.9	185.2	26.6	32.4	20.3	206.5	207.6	26.1	32.5	21.4	205.7	218.9	26.4	33.2	21.6	202.3	226.5
	R1 to R3	28.4	35.9	22.7	444.6	405.6	28.1	35.7	23.0	445.1	394.7	28.2	35.8	23.2	428.8	375.9	28.4	35.3	23.3	423.1	357.4
	R3 to R6	26.1	30.1	18.1	533.5	633.8	26.3	30.6	18.6	542.9	573.7	26.2	30.5	18.3	530.6	546.9	26.4	31.3	18.9	545.6	506.3
SD2	Sowing to V12	27.5	32.5	22.7	730.5	755.1	27.7	32.7	22.8	720.9	771.8	27.4	32.2	22.7	733.8	769.8	27.4	32.2	21.7	721.8	798.2
	V12 to R1	29.6	36.8	23.7	246.8	188.7	29.4	35.9	23.8	239.6	219.7	29.6	35.7	23.3	238.7	218.0	29.7	35.9	23.3	231.1	224.4
	R1 to R3	27.1	34.5	21.0	382.1	362.0	27.4	34.8	21.5	386.1	354.7	27.6	33.3	22.0	381.2	342.0	27.5	34.8	22.5	387.9	324.6
	R3 to R6	22.5	26.9	16.1	460.8	602.1	22.7	27.5	16.2	472.2	536.8	22.6	27.8	15.7	469.9	495.5	22.9	28.4	16.1	477.7	449.7
SD3	Sowing to V12	28.7	34.7	23.7	746.6	692.2	28.7	34.6	23.8	736.4	700.5	28.1	34.8	23.5	763.0	727.3	28.0	34.7	23.5	730.4	748.1
	V12 to R1	28.4	34.3	22.1	222.2	210.2	28.2	34.1	22.5	210.7	237.2	28.2	34.3	22.5	202.8	222.8	28.1	34.3	22.7	210.9	232.4
	R1 to R3	24.1	29.0	17.2	308.2	350.5	23.9	28.9	17.3	308.6	341.7	24.3	30.4	17.3	307.1	333.1	24.2	30.6	17.4	309.8	312.2
	R3 to R6	17.2	22.9	11.8	313.2	581.2	17.6	23.2	12.3	350.7	529.5	17.4	21.9	11.4	319.0	482.9	17.7	22.4	11.9	344.8	435.4

SD1, early sowing; SD2, conventional sowing; SD3, delay sowing. V6, 6-leaf stage; V12, 12-leaf stage; R1, silking stage; R3, milking stage; R6, physiological maturity. T_mean_, daily mean temperature; T_max_, daily maximum temperature; T_min_, daily minimum temperature; AT, effective accumulated temperature; R_a_, accumulated radiation.

The changes in temperature and radiation conditions contributed to the variation in phenological stages of maize (Table 2). Compared to SD2, SD1 increased the growth period before silking and after silking by 4.8 and 4.0 days, respectively, while SD3 decreased the growth period before silking by 3.3 days, and increased the growth period after silking by 9.5 days.

**TABLE 2:** Dates and days of maize phenology under different sowing dates in 2019 and 2020.

Year	Variety	Sowing date	Date						
			
			Sowing	VE	V6	V12	R1	R3	R6
2019	ZD958	SD1	5/21	5/28 (7)	6/18 (28)	7/7 (47)	7/20 (60)	8/12 (83)	9/23 (125)
		SD2	6/10	6/16 (6)	7/5 (25)	7/23 (43)	8/4 (55)	8/25 (76)	10/4 (116)
		SD3	6/30	7/5 (5)	7/23 (23)	8/9 (40)	8/21 (52)	9/12 (74)	10/30 (122)
	XY335	SD1	5/21	5/29 (8)	6/18 (28)	7/6 (46)	7/18 (58)	8/10 (81)	9/21 (123)
		SD2	6/10	6/16 (6)	7/4 (24)	7/22 (42)	8/2 (53)	8/23 (74)	10/1 (113)
		SD3	6/30	7/5 (5)	7/22 (22)	8/8 (39)	8/19 (50)	9/10 (72)	10/28 (120)
2020	ZD958	SD1	5/21	5/29 (8)	6/19 (28)	7/8 (48)	7/20 (60)	8/11 (82)	9/22 (124)
		SD2	6/10	6/16 (6)	7/5 (25)	7/23 (43)	8/5 (56)	8/26 (77)	10/5 (117)
		SD3	6/30	7/5 (5)	7/23 (23)	8/10 (41)	8/22 (53)	9/13 (75)	10/31 (123)
	XY335	SD1	5/21	5/29 (8)	6/18 (28)	7/7 (47)	7/18 (58)	8/9 (80)	9/20 (122)
		SD2	6/10	6/16 (6)	7/4 (24)	7/23 (43)	8/3 (54)	8/24 (75)	10/2 (114)
		SD3	6/30	7/5 (5)	7/21 (21)	8/7 (38)	8/19 (50)	9/11 (73)	10/29 (121)

SD1, early sowing; SD2, conventional sowing; SD3, delay sowing. VE, the emergency stage; V6, 6-leaf stage; V12, 12-leaf stage; R1, silking stage; R3, milking stage; R6, physiological maturity.

### Malondialdehyde content and antioxidant enzyme activities of ear leaf

**FIGURE 1:**
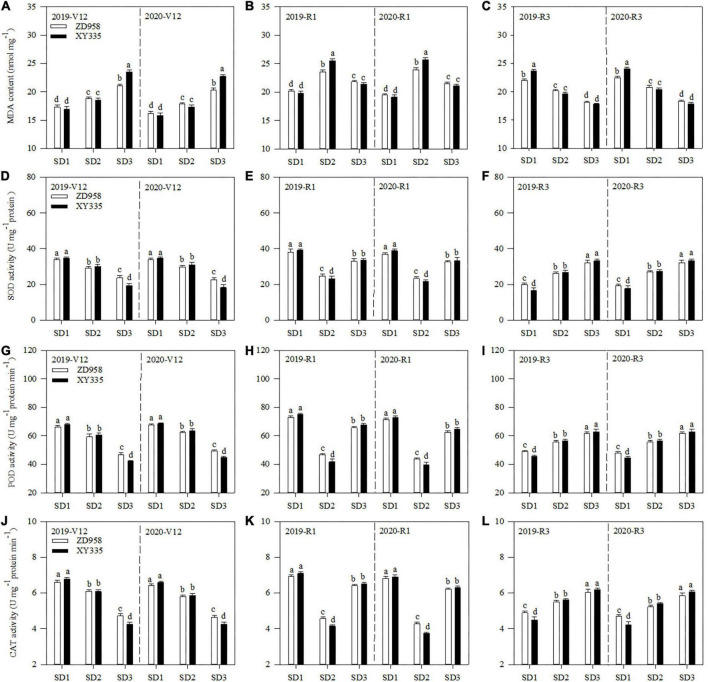
MDA content **(A–C)** and activities of SOD **(D–F)**, POD **(G–I)**, and CAT **(J–L)** of maize ear leaf at V12, R1, and R3 stages under different sowing dates in 2019 and 2020. SD1, early sowing; SD2, conventional sowing; SD3, delay sowing; POD, peroxidase; CAT, catalase; MDA, malondialdehyde; V12, 12-leaf stage; R1, silking stage; R3, milking stage. Different letters above the error bars indicate significant difference at the 0.05 probability level.

Both the sowing date and variety affected the MDA content and the SOD, POD, and CAT activities of the ear leaf (Figure 1). SD1 decreased the MDA content of ear leaf by 9.5 and 9.3% at V12, and 19.6 and 25.6% at R1, respectively, compared to SD2 across 2 years. Meanwhile, SD3 decreased the MDA content of ZD958 and XY335 by 9.7 and 21.6% at R1, and 12.5 and 12.1% at R3, respectively, compared to SD2 across 2 years.

Contrary to MDA content, SD1 increased the SOD, POD, and CAT activities of ZD958 by 14.8, 10.6, and 9.5% at V12, and 56.2, 59.7, and 55.5% at R1, while SD1 increased those of XY335 by 13.5, 10.4, and 11.9% at V12, and 73.7, 69.0, and 61.0% at R1, respectively, compared with SD2 across 2 years. Meanwhile, SD3 increased the SOD, POD, and CAT activities of ZD958 by 36.8, 41.7, and 42.8% at R1, and 20.7, 11.1, and 11.0% at R3, while SD3 increased those of XY335 by 48.8, 62.2, and 62.4% at R1, and 23.3, 10.9, and 11.2% at R3, respectively, compared with SD2 across 2 years.

### Photosynthetic parameters of ear leaf

**FIGURE 2:**
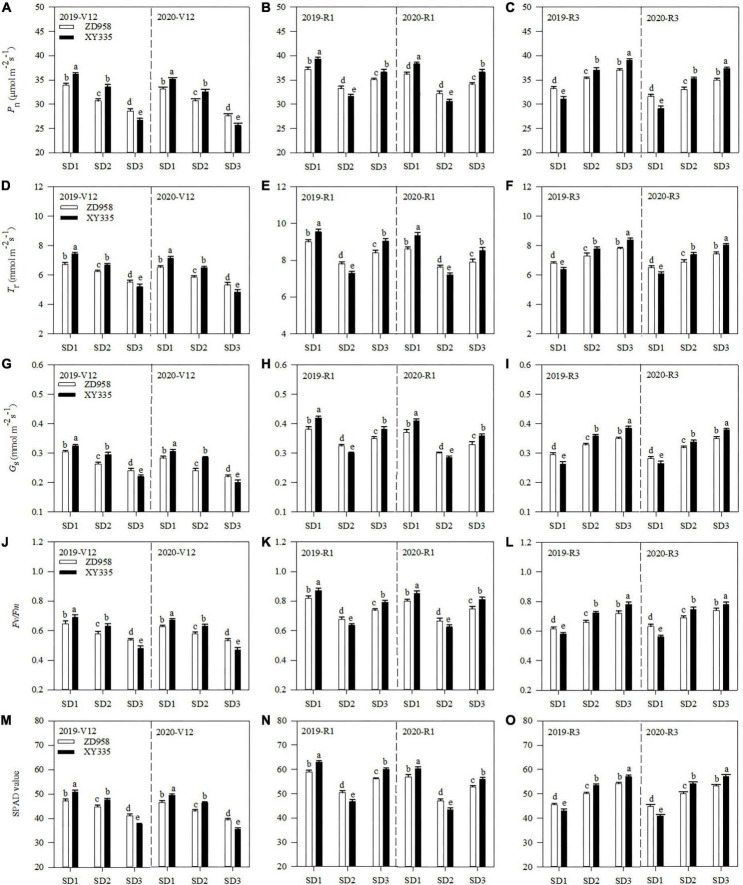
*P*_n_ **(A–C)**, *T*_r_ **(D–F)**, *G*_s_ **(G–I)**, *F*_v_*/F*_m_** (J–L)**, and SPAD **(M–O)** values of maize ear leaf at V12, R1, and R3 stages under different sowing dates in 2019 and 2020. SD1, early sowing; SD2, conventional sowing; SD3, delay sowing; *P*_n_, photosynthetic rate; *T*_r_, transpiration rate; *G*_s_, stomatal conductance; *F*_v_*/F*_m_, chlorophyll fluorescence parameters; V12, 12-leaf stage; R1, silking stage; R3, milking stage. Different letters above the error bars indicate significant difference at the 0.05 probability level.

*P*_n_, *T*_r_, and *G*_s_ varied by sowing dates and varieties (Figures 2A–I). Under SD1, *P*_n_, *T*_r_, and *G*_s_ of ZD958 increased by 9.3, 11.2, and 16.8% at V12, and 12.2, 14.2, and 20.3% at R3, while those of XY335 increased by 7.9, 10.6, and 8.7% at V12, and 24.8, 30.3, and 37.2% at R3, than those under SD2 across 2 years, respectively. Under SD3, *P*_n_, *T*_r_, and *G*_s_ of ZD958 increased by 5.9, 5.8, and 8.7% at R1, and 5.4, 7.2, and 9.7% at R3, while those of XY335 increased by 17.7, 21.3, and 22.5% at R1, and 5.7, 8.4, and 9.7% at R3, than those under SD2 across 2 years, respectively.

The *F*_v_*/F*_m_ and SPAD value also varied by sowing dates and varieties (Figures 2J–O). Compared with SD2, the *F*_v_*/F*_m_ and SPAD value of ZD958 under SD1 increased by 9.8 and 6.7% at V12, and 20.6 and 18.9% at R3, while those of XY335 increased by 8.1 and 6.8% at V12, and 236.4 and 36.8% at R3 across 2 years, respectively. Meanwhile, the *F*_v_*/F*_m_ and SPAD value of ZD958 under SD3 increased by 10.6 and 11.6% at R1, and 8.1 and 7.3% at R3, while those of XY335 increased by 27.1 and 28.6% at V12, and 6.1 and 5.9% at R3 across 2 years, respectively.

### Dry matter accumulation in plants

**FIGURE 3:**
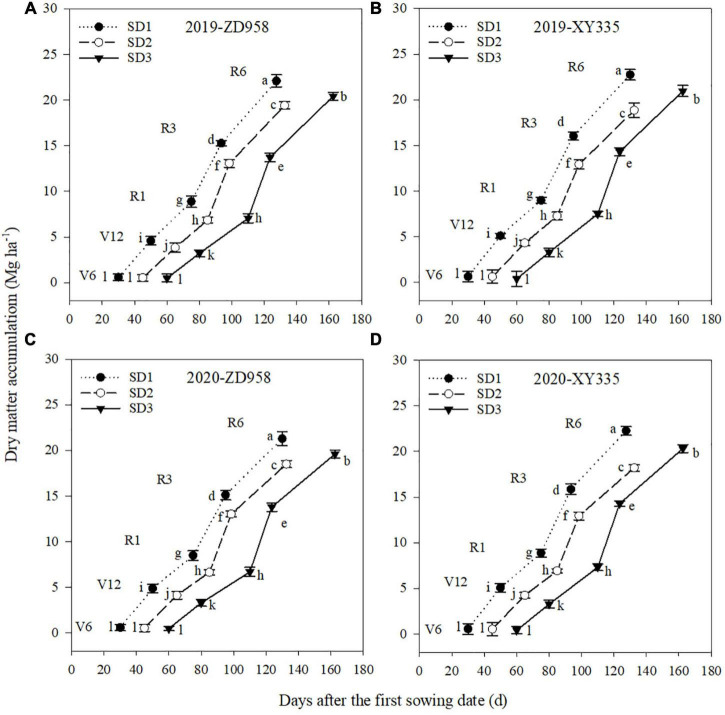
Dry matter accumulation of maize for each growth stage under different sowing dates in 2019 **(A,B)** and 2020 **(C,D)**.

The DM accumulation varied by sowing dates, varieties, and growth stages (Figure 3). Under SD1, the DM accumulation of ZD958 and XY335 averagely increased by 18.7 and 18.8% from V6 to V12 stages, and 43.4 and 36.2% from V12 to R1 stages, compared to SD2, respectively. Under SD3, the DM accumulation of ZD958 and XY335 averagely increased by 9.5 and 18.3% from R1 to R3 stages, and 5.6 and 13.2% from R3 to R6 stages, than that under SD2, respectively. Therefore, early sowing significantly increased maize DM accumulation pre-silking, while delay sowing significantly increased maize DM accumulation post-silking for both varieties in both years. Meanwhile, in both years, the DM of XY335 at V12, R1, R3, and R6 stages was greater than that of ZD958 under SD1 and SD3, while no significant variation was found between ZD958 and XY335 under SD2.

### Kernel number, kernel weight, and grain yield

**FIGURE 4:**
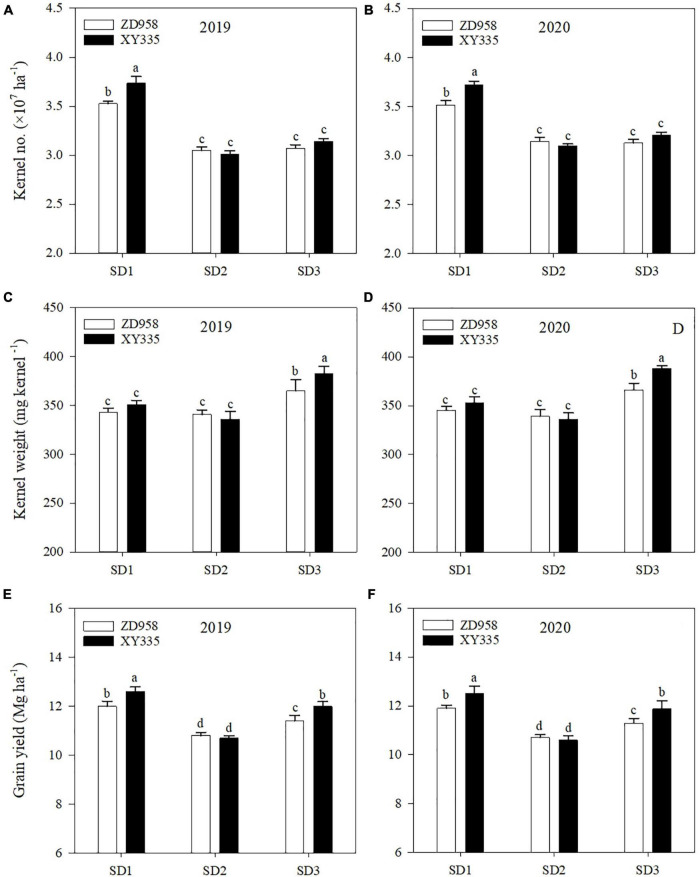
Kernel number **(A,B)**, kernel weight **(C,D)**, and grain yield **(E,F)** of maize under different sowing dates in 2019 and 2020. SD1, early sowing; SD2, conventional sowing; SD3, delay sowing. Different letters above the error bars indicate significant difference at the 0.05 probability level.

The kernel number, kernel weight, and grain yield were also affected by sowing date and variety (Figure 4). SD1 averagely increased the kernel number of ZD958 and XY335 by 13.7 and 22.1%, than that of SD2 in 2 years, respectively. However, SD3 averagely increased the kernel weight of ZD958 and XY335 by 7.5 and 14.7% in 2 years, respectively. Therefore, the grain yield of ZD958 and XY335 averagely increased by 11.2 and 17.8% under SD1, and 5.6 and 12.2% under SD3 in 2 years, compared to SD2, respectively. Meanwhile, XY335 revealed a greater kernel number under SD1 and greater kernel weight under SD3 than ZD958, and the grain yield of XY335 was greater than that of ZD958 under SD1 and SD3 in both years.

As shown in Table 3, kernel number was positively correlated with *P*_n_, *T*_r_, *G*_s_, *Fv/Fm*, SPAD, MDA, SOD, POD, and CAT at V12 and R1 stages, kernel weight was correlated with those physiological parameters at the R3 stage, and grain yield was correlated with those physiological parameters at the R1 stage.

**TABLE 3:** Relationship between physiological parameters and yield traits at different sowing dates.

Items	Growth stage	*P*_n_	*T*_r_	*G*_s_	*Fv/Fm*	SPAD	MDA	SOD	POD	CAT
Kernel number	V12	0.646*	0.660*	0.461	0.694*	0.584*	–0.583*	0.689*	0.601*	0.603*
	R1	0.809**	0.816**	0.635*	0.856**	0.726**	–0.795**	0.781**	0.742**	0.701*
Kernel weight	R1	0.451	0.400	0.508	0.369	0.497	–0.416	0.455	0.487	0.426
	R3	0.611*	0.607*	0.619*	0.584*	0.584*	–0.594*	0.578*	0.598*	0.589*
Grain yield	V12	0.215	0.264	0.093	0.280	0.149	–0.060	0.227	0.115	0.130
	R1	0.984**	0.977**	0.878**	0.952**	0.960**	–0.925**	0.955**	0.943**	0.927**
	R3	–0.316	–0.327	–0.367	–0.411	–0.433	0.423	–0.476	–0.431	–0.422

V12, 12-leaf stage; R1, silking stage; R3, milking stage. * Significant at the 0.05 probability level. ** Significant at the 0.01 probability level.

### Relationship between climatic factors and kernel number, kernel weight, and grain yield

Correlation and regression analyses were performed between climatic factors and kernel number, kernel weight, and grain yield of maize (Table 4). Negative correlations were found between kernel number and *T*_mean_, *T*_max_, and *T*_min_ from V12 to R1 stages, between kernel weight and *T*_mean_, *T*_max_, *T*_min_, and AT from R1 to R3 stages, and between grain yield and *T*_mean_, *T*_max_, *T*_min_, and AT from V12 to R1 stages. There were no significant correlations between radiation and kernel number, kernel weight, and grain yield at each stage.

**TABLE 4:** Relationships between temperatures during maize growth stage and kernel number, kernel weight, and grain yield.

Items	Growth stage	*T*_mean_	*T*_max_	*T*_min_	AT	*R*_a_
Kernel number	Sowing to V12	–0.246	–0.287	–0.295	–0.126	0.378
	V12 to R1	–0.816**	–0.740**	–0.878**	–0.118	–0.375
	R1 to R3	0.242	0.324	0.302	0.252	0.411
	R3 to R6	0.325	0.312	0.232	0.234	0.440
Kernel weight	Sowing to V12	0.325	0.249	0.297	0.016	–0.324
	V12 to R1	–0.103	–0.223	0.160	0.096	–0.134
	R1 to R3	–0.874**	–0.753**	–0.748**	–0.618*	–0.262
	R3 to R6	–0.256	–0.253	–0.248	–0.313	–0.193
Grain yield	Sowing to V12	–0.533	–0.223	–0.506	–0.441	0.296
	V12 to R1	–0.903**	–0.892**	–0.844**	–0.850**	–0.370
	R1 to R3	0.057	0.063	0.083	0.262	0.249
	R3 to R6	0.276	0.246	0.256	0.221	0.380

V6, 6-leaf stage; V12, 12-leaf stage; R1, silking stage; R3, milking stage; R6, physiological maturity. T_mean_, daily mean temperature; T_max_, daily maximum temperature; T_min_, daily minimum temperature; AT, effective accumulated temperature; R_a_, accumulated radiation.

*Significant at the 0.05 probability level. **Significant at the 0.01 probability level.

**FIGURE 5:**
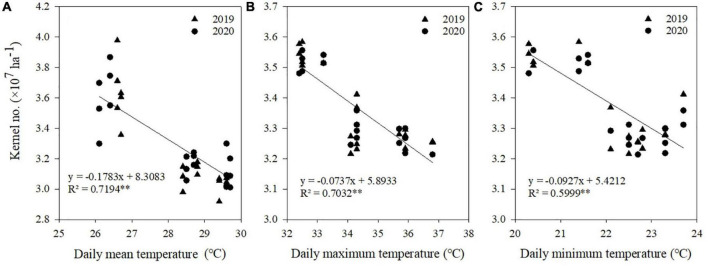
Relationships between kernel number and daily mean temperature **(A)**, daily maximum temperature **(B)**, and daily minimum temperature **(C)** from V12 to R1. **Significant at the 0.01 probability level.

**FIGURE 6:**
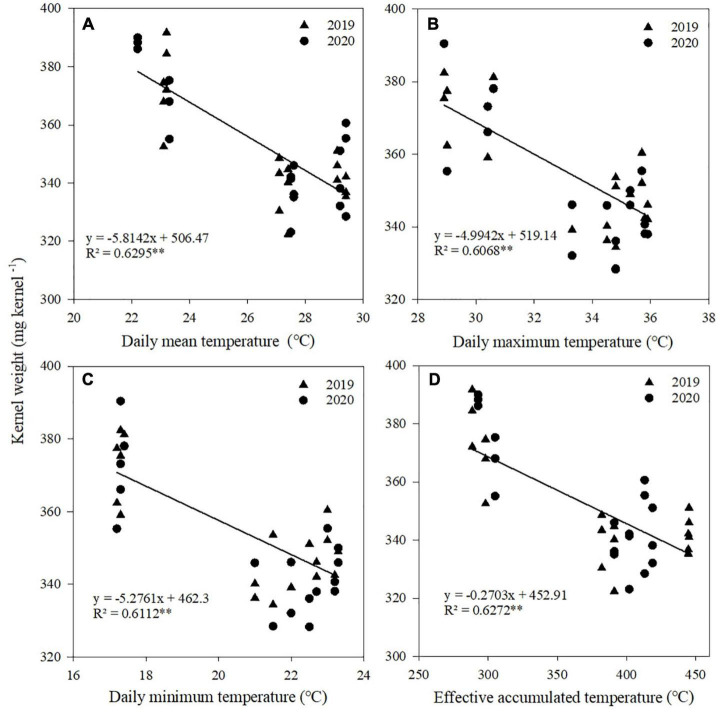
Relationships between kernel weight and daily mean temperature **(A)**, daily maximum temperature **(B)**, daily minimum temperature **(C)**, and effective accumulated temperature **(D)** from R1 to R3. **Significant at the 0.01 probability level.

**FIGURE 7:**
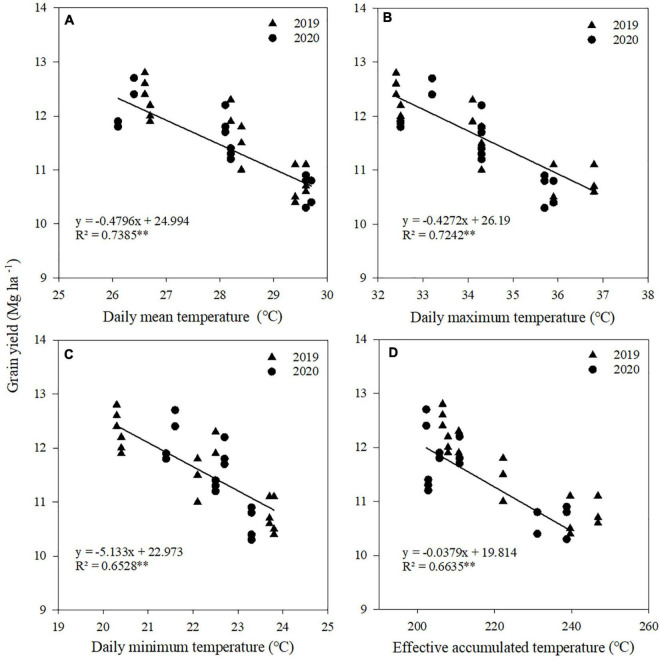
Relationships between grain yield and daily mean temperature **(A)**, daily maximum temperature **(B)**, daily minimum temperature **(C)**, and effective accumulated temperature **(D)** from V12 to R1. **Significant at the 0.01 probability level.

A significant linear relationship existed between kernel number and *T*_mean_, *T*_max_, and *T*_min_ from V12 to R1 stages (Figure 5), between kernel weight and *T*_mean_, *T*_max_, *T*_min_, and AT from R1 to R3 stages (Figure 6), and between grain yield and *T*_mean_, *T*_max_, *T*_min_, and AT from R1 to R3 stages (Figure 7). When *T*_mean_, *T*_max_, and *T*_min_ from V12 to R1 stages were higher than 26.6, 32.5, and 20.3°C, and *T*_mean_, *T*_max_, *T*_min_, and AT from R1 to R3 stages were higher than 23.2, 28.9, 17.3, and 288.6°C, respectively, the kernel number, kernel weight, and grain yield decreased.

## Discussion

Previous studies have demonstrated that adjusting the sowing date is an effective way to avoid the adverse effects of climate conditions on maize growth by changing the climatic factors during the maize growth period (Zhang et al., 2020; Coelho et al., 2021; Tian et al., 2021). Our results showed that compared to conventional sowing (SD2), early sowing (SD1) markedly decreased *T*_mean_, *T*_max_, and *T*_min_ during pre-silking (especially from V12 to R1 stages), while delay sowing (SD3) markedly decreased those temperatures during post-silking (especially from R1 to R3 stages), which are closely related to the grain formation and yield of maize (Badu–Apraku et al., 1983). Therefore, SD1 and SD3 averagely increased the maize yield by 11.2 and 17.8% compared to SD2, respectively. Yield decrease for SD2 due to high temperature during flowering was associated with a reduction in kernel number and kernel weight. The kernel number of SD1 and kernel weight of SD3 were greater than that of SD2 for both varieties in both years, respectively. This finding could be confirmed by previous studies that high temperature could significantly affect the kernel number and kernel weight of maize (Borrás and Gambín, 2010; Zhang et al., 2019).

Photo-assimilated carbon as the primary factor in determining maize yield generally decreases with increasing temperature (Roger et al., 1983; Yan et al., 2017; Zheng et al., 2018). In this study, high temperatures (*T*_mean_, *T*_max_, and *T*_min_) occurred from V12 to R3 stages under the SD2 condition, which was greater than that for SD1 from V12 to R1 stages, and for SD3 from R1 to R3 stages. High-temperature stress could increase the number of reactive oxygen species (ROS) such as superoxide anion radical (O_2_^–^), H_2_O_2_, and hydroxyl radical (OH^–^) (Liu and Huang, 2000; Barnabas et al., 2008) and then result in the membrane peroxidation and accumulation of MDA (Xu et al., 2006). Moreover, although high-temperature stress could increase the activities of SOD, POD, and CAT to remove sufficient ROS (Blokhina et al., 2003; Obata et al., 2015), the activities of these enzymes decreased with an increase in the frequency of extreme high-temperature events (Dat et al., 1998; Doru, 2021). Therefore, maize under SD2 had greater MDA content with lower activity of SOD, POD, and CAT in maize ear leaf compared to SD1 at V12 and R1 stages, and SD3 at R1 and R3 stages. This could damage the structure of a photosynthetic system and reduce the electron transport efficiency and chlorophyll content (Prasad et al., 2011), thus decreasing the photosynthetic rate (Veerasamy et al., 2007) and the assimilate capacity of a leaf (Sinsawat et al., 2004; Rattalino et al., 2014). However, the lower temperatures for SD1 from V12 to R1 stages, while for SD3 from R1 to R3 stages compared to SD2, promoted maize leaf to maintain higher antioxidant capacity (e.g., lower MDA and higher activities of SOD, POD, and CAT) and photosynthetic activity (e.g., higher SPAD, *F*_v_*/F*_m_, *P*_n_, *T*_r_, and *G*_s_). As a result, the pre-silking DM accumulation for SD1 and the post-silking DM accumulation for SD3 averagely increased by 17.9 and 8.4% compared to SD2, respectively. Greater pre-silking DM accumulation of maize had an effect on developing kernels, with greater translocation of DM to help establish viable embryos (He et al., 2005; Yan et al., 2017; Yu et al., 2021), while greater post-silking DM accumulation improved the capacity of maize to provide assimilates during grain filling (Echarte et al., 2008; Ning et al., 2013; Paponov et al., 2020). Moreover, optimal temperature condition could increase the activities of key enzymes in carbon metabolism of grain, and promote the transport, transformation, and accumulation of assimilates into grains (Suwa et al., 2010). Consequently, kernel number of SD1 and kernel weight of SD3 for both varieties significantly increased in both years, which contributed to increase the yield of SD1 and SD3 compared to SD2.

Previous studies have suggested that *T*_mean_ of 24–26°C is required for maize flowering to achieve a high yield of maize (Xu et al., 2021). *T*_max_ of more than 32°C during the pre-silking stage reduces the kernel number, while *T*_max_ of more than 25°C during the grain-filling stage decreases the kernel weight (Wilhelm et al., 1999). Our results showed that the kernel number was negatively correlated with *T*_mean_, *T*_max_, and *T*_min_ from V12 to R1 stages, while the kernel weight was negatively correlated with *T*_mean_, *T*_max_, *T*_min_, and AT from R1 to R3 stages. The results of regression analysis indicated that high temperatures (*T*_mean_ > 26.6°C, *T*_max_ > 32.5°C, and *T*_min_ > 20.3°C) from V12 to R1 stages could decrease the kernel number, while high temperatures (*T*_mean_ > 23.2°C, *T*_max_ > 28.9°C, *T*_min_ > 17.3°C, and AT > 288.6°C) from R1 to R3 stages could decrease the kernel weight, eventually decreasing the grain yield. These findings were consistent with earlier studies that high-temperature stress during the 9th leaf stage to the tasseling stage of maize could decrease the grain number at the ear tip (Shao et al., 2021), while high-temperature stress during the grain filling stage could lower the filling rate and reduce the kernel weight (Tao et al., 2016). Therefore, in our study, SD1 had greater kernel number and SD3 had greater kernel weight compared to SD2 due to optimized temperature condition around flowering. However, Tian et al. (2018) found that late sowing of maize could lead to the late grain filling phase coincided with decreasing temperature (average minimum temperature was 11.3 and 12.9°C 5 and 10 days before maturity, respectively), thus decreasing the grain filling rate. As we know, DM accumulation in kernels depends on both kernel growth rate and duration of grain filling (Borrás and Gambín, 2010), and lower temperature decreases grain filling rate while extending grain filling duration (Jones et al., 1981). In our study, the average minimum temperature in SD3 was lower than 12°C from R3 to R6 stages, which may decrease the grain filling rate, but the duration of grain filling (after silking) in SD3 increased by 9.5 days compared with SD2. Several studies also demonstrate that the solar radiation and rainfall are also limiting factors for kernel weight of late sowing maize (Gao et al., 2018; Li et al., 2022). In our study, the maize was fully irrigated in the field experiment, and no obvious water stress was observed during the growing season. Meanwhile, SD3 decreased the radiation from R1 to R3 and R3 to R6 only by 3.4 and 2.7% compared to SD2, and by 12.8 and 10.4% compared to SD1 across years and varieties, respectively, which might decrease the production of photosynthate post-silking (Gao et al., 2018). However, a previous study has demonstrated that 19–34% of the grain yield maize was from remobilization of the reserved carbon pre-silking (He et al., 2005), while the increased duration of grain filling of SD3 could provide more time for remobilization of DM pre-silking to increase the kernel weight. We concluded that temperature conditions around flowering of summer maize with early sowing (21 May) or delay sowing (30 June) in the NCP are suitable for kernel growth and a relatively high maize yield can be obtained.

Obviously, under global climate change conditions, adjusting the sowing date could be considered an effective way to avoid the adverse effects of high temperature on maize growth by changing the temperature conditions during the growing period of maize. However, maize hybrid with different maturations also could be chosen to make the crop growth match well with the changing climatic conditions. This study is an important future research priority for us.

## Conclusion

Early sowing markedly decreased the daily mean temperature (*T*_mean_), maximum temperature (*T*_max_), and minimum temperature (*T*_min_) during pre-silking, while delay sowing markedly decreased those temperatures during post-silking. Thus, maize of SD1 at V12 and R1 stages while SD3 at R1 and R3 stages showed lower MDA content and higher activities of CAT and POD, and higher SPAD, *F*_v_*/F*_m_, *P*_n_, *T*_r_, and *G*_s_. As a result, SD1 accumulated greater pre-silking DM to increase the kernel number, while SD3 accumulated greater post-silking DM to increase the kernel weight, eventually increasing the grain yield of SD1 and SD3 compared to SD2. We concluded that early sowing (21 May) or delay sowing (30 June) could optimize the temperature conditions around the flowering of summer maize to improve kernel growth and obtain a relatively higher maize yield in the NCP.

## Data availability statement

The original contributions presented in the study are included in the article/supplementary material, further inquiries can be directed to the corresponding author/s.

## Author contributions

BZ, ML, and MZ conceived and designed the experiment. DG, CC, XL, and RW carried out the experiments and performed the statistical analyses. DG, CC, and BZ drafted the manuscript. ZD, WM, XW, and CL reviewed and edited the manuscript. All authors contributed to the article and approved the submitted version.
